# Dendritic Cells from HIV Controllers Have Low Susceptibility to HIV-1 Infection *In Vitro* but High Capacity to Capture HIV-1 Particles

**DOI:** 10.1371/journal.pone.0160251

**Published:** 2016-08-09

**Authors:** Chiraz Hamimi, Annie David, Pierre Versmisse, Laurence Weiss, Timothée Bruel, David Zucman, Victor Appay, Arnaud Moris, Marie-Noëlle Ungeheuer, Caroline Lascoux-Combe, Françoise Barré-Sinoussi, Michaela Muller-Trutwin, Faroudy Boufassa, Olivier Lambotte, Gianfranco Pancino, Asier Sáez-Cirión, ANRS CO21 CODEX cohort

**Affiliations:** 1 Institut Pasteur, Régulation des Infections Rétrovirales, Paris, France; 2 Institut Pasteur, HIV Inflammation et Persistance, Paris, France; 3 AP-HP Hôpital Européen Georges Pompidou, Paris, France; 4 Université Paris Descartes; Sorbonne Paris-Cité; Paris, France; 5 Université Paris Sud, UMR-1184, Le Kremlin-Bicêtre, France; 6 CEA, DSV/iMETI, Division of Immuno-Virology, IDMIT, Fontenay-aux-Roses, France; 7 Hopital Foch, Service de médecine interne, Suresnes, France; 8 Sorbonne Universités, UPMC Univ Paris 06, DHU FAST, CR7, Centre d’Immunologie et des Maladies Infectieuses (CIMI-Paris), Paris, France; 9 INSERM, U1135, CIMI-Paris, Paris, France; 10 CNRS, ERL 8255, CIMI-Paris, Paris, France; 11 Institut Pasteur, Plate-forme Investigation Clinique et Accès aux Ressources Biologiques (ICAReB), Paris, France; 12 AP-HP, Saint-Louis Hospital, Infectious Diseases Department, Paris, France; 13 INSERM U1018, Faculté de Médecine Paris Sud, Le Kremlin-Bicêtre, France; 14 Inserm, U1184, Center for immunology of viral infections and autoimmune diseases, Le Kremlin-Bicêtre, France; 15 APHP, Hôpitaux Universitaires Paris Sud, Service de Médecine Interne–Immunologie Clinique, le Kremlin Bicêtre, France; Instituto de Salud Carlos III, SPAIN

## Abstract

HIV controllers (HICs), rare HIV-1 infected individuals able to control viral replication without antiretroviral therapy, are characterized by an efficient polyfunctional and cytolytic HIV-specific CD8+ T cell response. The mechanisms underlying the induction and maintenance of such response in many HICs despite controlled viremia are not clear. Dendritic cells play a crucial role in the generation and reactivation of T cell responses but scarce information is available on those cells in HICs. We found that monocyte derived dendritic cells (MDDCs) from HICs are less permissive to HIV-1 infection than cells from healthy donors. In contrast MDDCs from HICs are particularly efficient at capturing HIV-1 particles when compared to cells from healthy donors or HIV-1 patients with suppressed viral load on antiretroviral treatment. MDDCs from HICs expressed on their surface high levels of syndecan-3, DC-SIGN and MMR, which could cooperate to facilitate HIV-1 capture. The combination of low susceptibility to HIV-1 infection but enhanced capacity to capture particles might allow MDDCs from HICs to preserve their function from the deleterious effect of infection while facilitating induction of HIV-specific CD8+ T cells by cross-presentation in a context of low viremia.

## Introduction

HIV controllers (HIC), rare HIV-1 infected individuals, are able to maintain undetectable viremia for several years without any therapeutic intervention [1, 2]. Several studies reported the major role of CD8+ T cell response in viral containment in these patients. Indeed, despite low viremia, HICs have high frequency of HIV-specific CD8+ T cells [3, 4] displaying a more polyfunctional response to HIV than cells from patients non-controlling infection [5, 6]. Moreover, CD8+ T cells from HIC exhibit a striking capacity to eliminate autologous infected CD4+ T cells [4, 7], which is likely related to their capacity to rapidly upregulate cytotoxic granules [8, 9]. The presence of “protective” HLA alleles, such as HLA-B*57 or B*27, which are overrepresented in HIC [4, 10, 11], may contribute to the priming of such effective HIV-specific CD8+ T cell responses by efficiently presenting HIV antigens and selecting high avidity CTL clonotypes [12, 13]. However, not all HLA-B*57 individuals are able to develop similar HIV-specific CD8+ T cell responses and many HIC with robust CD8+ T cell responses do not carry protective HLA alleles [3, 13, 14]. Therefore, understanding the mechanisms underlying the generation and the maintenance of such efficient CD8+ T cell response is of outmost interest, as this characterization might give insights for new therapeutic strategies to achieve control of infection in the absence of antiretroviral treatment.

Myeloid dendritic cells (DC) play a central role in the induction of virus-specific CD8+ T cell responses, since they are the most potent antigen presenting cells and unique for their capacity to activate naïve T cells. DCs activate CD8+ T cells by presenting antigen bound by major histocompatibility complex molecules class-I (MHC-I). Virus-infected DCs can use endogenously synthesized viral proteins stemming from viral replication as antigens for presentation on MHC-I and this process is commonly called “classic or direct presentation” [15]. Whereas, non-infected DCs need to actively engulf exogenous viral antigens for presenting them to CD8+ T cells by “cross-presentation” process [16].

DCs are susceptible to HIV-1 infection [17] although less permissive compared to activated CD4+ T cells. This restriction is related, in part, to the antiviral activity of SAMHD-1 that degrades the cellular dNTP pool [18, 19], crucial for HIV reverse transcription, and viral nucleic acids [20]. Restriction of HIV-1 replication in DCs prevents sensing of the virus [18, 21]. Indeed, when restriction is relieved by delivery of Vpx which induce SAMHD-1 degradation, there is better sensing of HIV-1 marked by induction of IFN-I response, up regulation of cell surface CD80 and CD86 costimulatory molecules that provide an efficient stimulation of CD4+ and CD8+ T cells [21]. We and others have demonstrated that CD4+ T cells and macrophages from HIC are less susceptible to HIV-1 infection than cells from other patients [22–24]. However it is unknown whether this resistance to HIV-1 also extends to HIC DCs. An analysis of the interactions between HIC DCs and HIV-1 appears thus crucial for better understanding the mechanisms underlying the priming and maintenance of the efficient HIV-specific CD8+ T cell response in HIC. In this context, we explored the susceptibility of HIC DCs to HIV-1 infection in vitro as well as their capacity to capture HIV-1 virions. Our findings indicate that DCs from HIC are relatively resistant to HIV-1 infection, which could preserve their function. In addition, DCs from HICs showed high capacity to uptake HIV-1 particles which might facilitate induction of HIV-specific CD8+ T cells by cross-presentation in the context of low viremia.

## Results

### MDDCs from HIC have low susceptibility to HIV-1 infection

**Fig 1:**
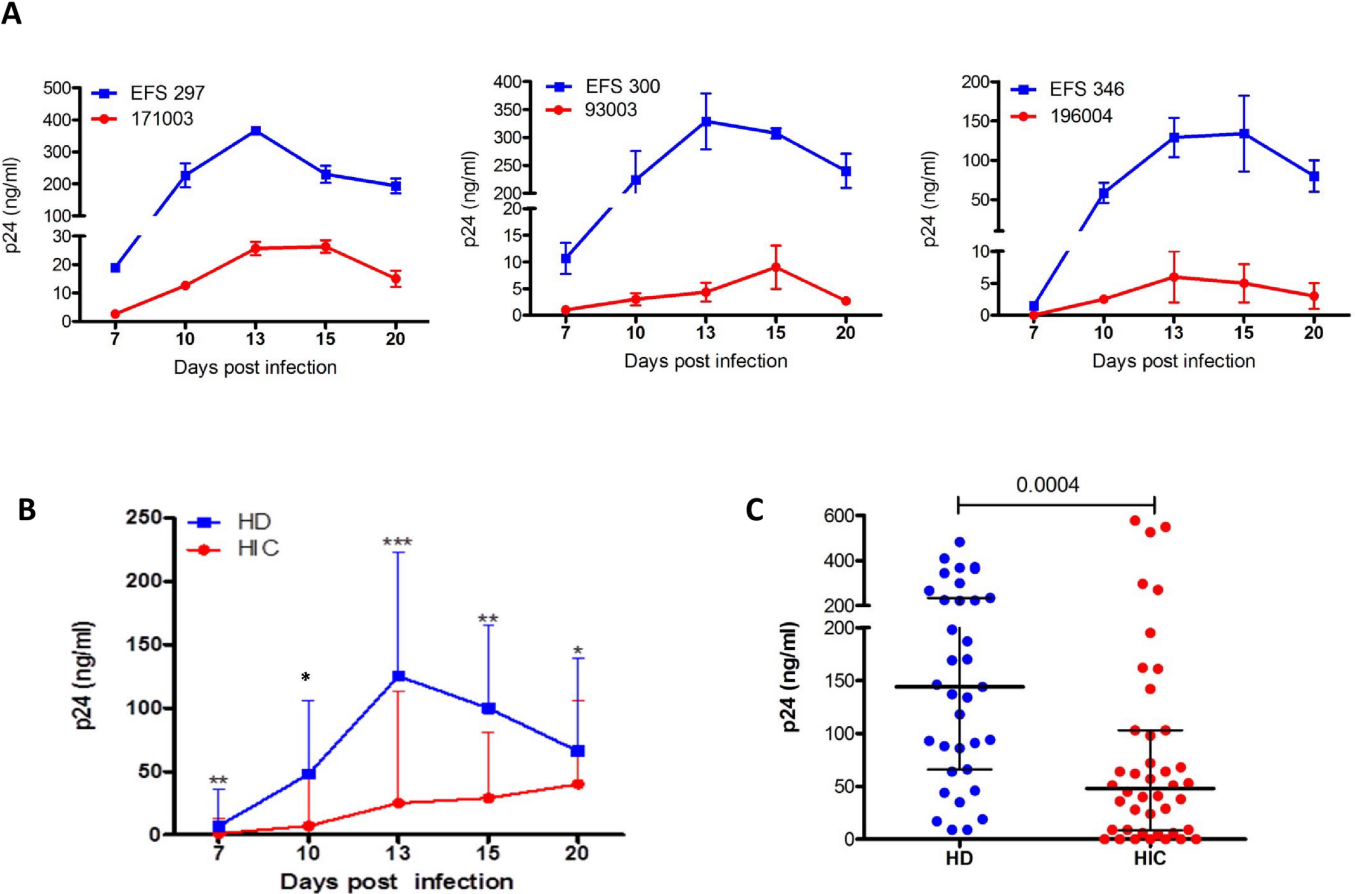
Susceptibility of MDDCs from HIC and HD to HIV-1 infection. **(A)** Kinetic of HIV-1 BaL replication in MDDC from three HIC (red) and three HD (blue) in three independent and representative examples. p24 production in culture supernatants is represented as the mean ± SD of 3 independent cultures for each individual. **(B)** Kinetic of HIV-1 Bal production in supernatant of HIC and HD MDDC’s. p24 production in culture supernatants is represented as the mean ± IQR of 34 HD and 42 HIC. (* p<0.05; ** p<0.01; *** p<0.001) **(C)** Viral production at peak of infection in culture supernatants. Symbols represent the average (n = 3 independent experiments) of p24 values detected in culture supernatants for each subject (HD n = 34 and HIC n = 42). Horizontal lines represent median ± interquartile values for each group.

We previously demonstrated that CD4+ T cells and macrophages from HIC are relatively resistant to HIV-1 infection [22]. We wondered whether the same resistance to infection could be found in DCs. Limited sample availability did not allow us to conduct functional experiments with circulating DCs. We therefore investigated the susceptibility to HIV-1 infection using monocytes derived dendritic cells (MDDC) from HICs (n = 42) and HDs (n = 34). MDDC were infected with HIV-1 BaL and cultured for 20 days post-infection. Viral production was monitored every 3–4 days. MDDCs from HDs sustained viral replication with peak viral production at days 13–15 post-infection (see Fig 1A for three representative experiments and Fig 1B for group medians). In contrast, viral replication was severely impaired in MDDCs from HICs at all time points analyzed (Fig 1B). Overall, the peak of viral replication was lower in MDDCs from HIC than from HD (p = 0.0004) (Fig 1C), demonstrating that HIV-1 replicates less efficiently in cells from HICs.

We wondered whether the reduced HIV-1 susceptibility of MDDCs from HICs to infection could be related to a stronger IFN-response in HICs’ MDDCs upon HIV-1 infection [21]. We measured the bioactive IFN-α in culture supernatants of MDDCs from 19 HICs and 15 HDs at 4h, 24h, 5, 7, 10 and 13 days after HIV-1 challenge. We found no difference between HIC and HD cells in the levels of IFN-α production. In fact, IFN-α was not detected in most samples and at most time points, with the exception of 3 HDs (EFS 171, EFS 300 and EFS 377, with 4.3, 5.4 and 16.5 IFN-α IU/ml, respectively) and one HIC (56011, 24 IFN-α IU/ml) at the peak of viral replication (S1 Table). Viral replication in the cells from these subjects was not different from what was observed with cells from other subjects in their groups. Overall, our results suggest that low HIV-1 susceptibility of HICs’ MDDCs is not related to an enhanced production of IFN-α by these cells upon contact with HIV-1.

### MDDCs from HICs have an enhanced capacity to capture HIV-1 particles

The reduced susceptibility of MDDCs from HICs to HIV-1 infection suggests that the superior HIV-specific CD8+ T cell responses found in HICs are not likely due to direct antigen presentation by their DCs. We wondered whether other mechanisms could help to an efficient presentation of HIV antigens to CD8+ T cells in the context of low productive infection. We therefore analyzed the capacity of MDDCs from HICs to capture free viral particles when compared to cells from HDs and cARTs.

**Fig 2:**
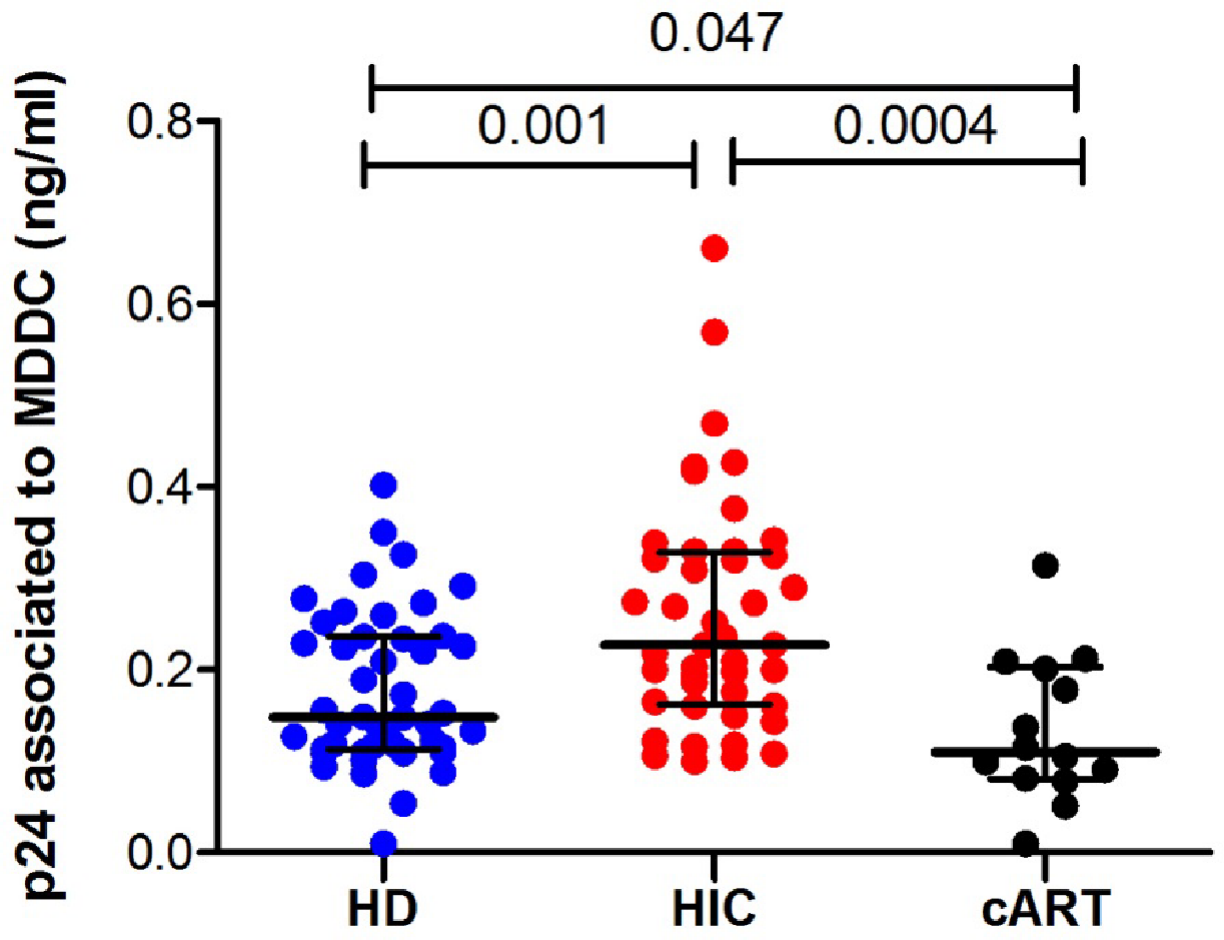
Capture of HIV-1 Bal by MDDC. Cell-associated p24 levels 4h after exposure to virus. Symbols represent the average p24 levels (n = 3 independent experiments) for each individual (HDs n = 47, HICs n = 44 and cARTs n = 14). Horizontal lines represent median ± interquartile values for each group.

MDDCs were incubated with HIV-1 BaL during 4h at 37°C. After extensive washing to get rid of unbound viral particles, cells were lysed and used to quantify the level of HIV-1 p24 antigens associated with the cells. Our results showed higher levels of p24 associated with MDDCs from HICs than with MDDCs from HDs (p = 0.001) or cART patients (p = 0.0004), the latter cells being the ones with the lowest levels of associated p24 (p = 0.047) (Fig 2). Interestingly, no correlation was found between the levels of HIV-1 capture and the susceptibility to HIV-1 infection of MDDCs from either HICs or HDs (S1 Fig). These results suggest that MDDCs from HICs captured HIV-1 particles more efficiently than cells from HDs and cARTs.

### MDDCs from HICs, HDs and cARTs have similar general capacities to capture and degrade antigens

We wondered whether the higher capacity of MDDCs from HIC to capture HIV-1 particles was related to a higher general capacity of their cells to capture and process antigens. We used Alexa 488-conjugated dextrans (10 000 MW) and DQ-ovalbumin to evaluate the capacity of MDDCs to uptake antigens by endocytosis through mannose receptors or by macropinocytosis. The DQ-Ovalbumin is a self-quenched BODIPY FL conjugate of albumin that exhibits bright fluorescence upon endo-lysosomal proteolysis, which also allowed us to evaluate the capacity of MDDCs to route antigens towards degradative compartments.

**Fig 3:**
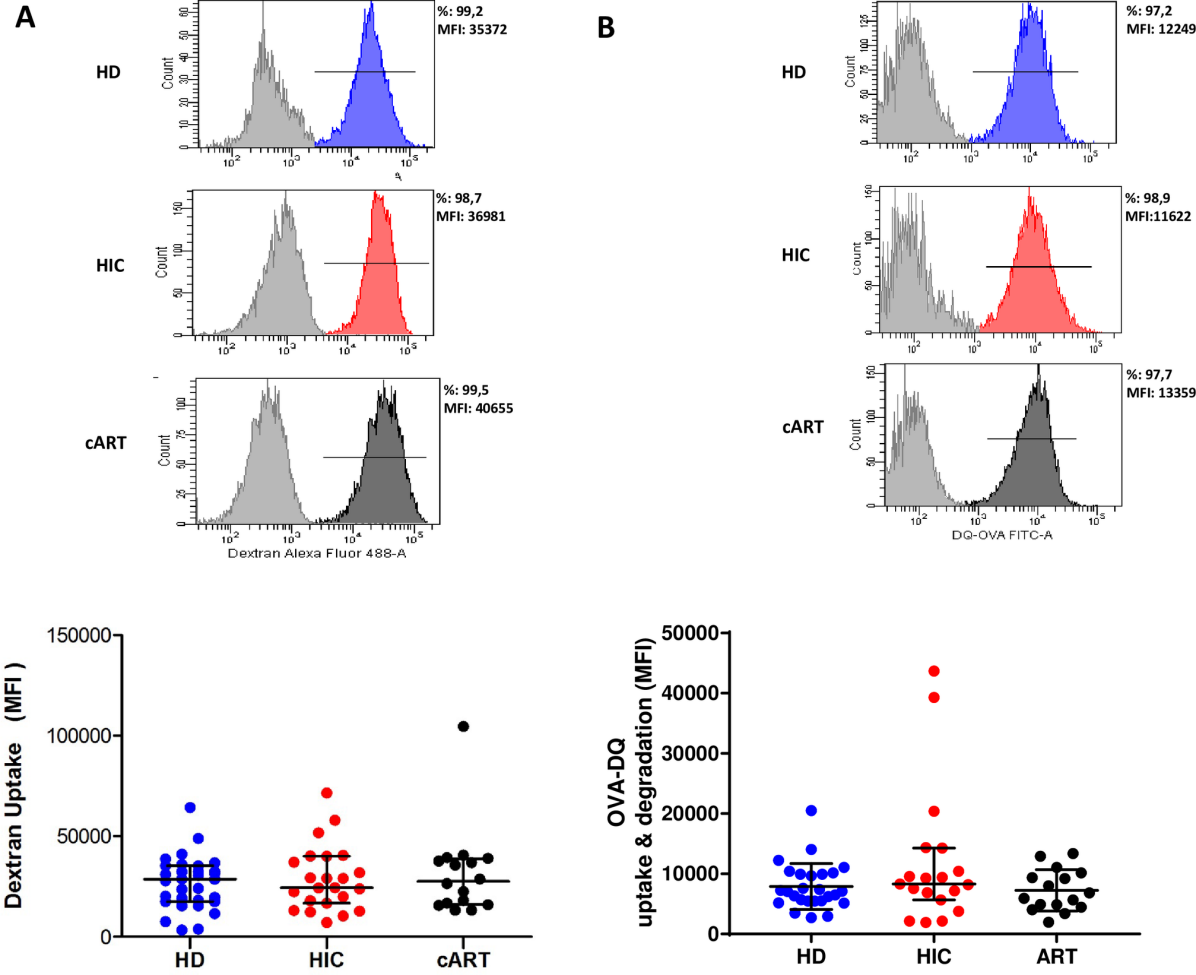
Antigen uptake and degradation by MDDC. Dextran uptake **(A)** and ovalbumine uptake and degradation **(B)** by MDDC from HDs, HICs and cARTs. Top panels are examples with cells from three representative individuals of each group (light grey is the negative control, blue is one HD, red one HIC and dark grey is a cART). Bottom panels present the summary of all the experiments performed. Each circle represents one individual, and the horizontal lines represent the median ± interquartile values for each group (HD n = 31, HIC n = 23 and cART n = 16).

Antigens were added to cell cultures and their uptake by MDDCs was measured 4h later by flow cytometry. As illustrated in Fig 3A, no differences were observed in the uptake capacity of MDDCs from HICs, HDs and cARTs. Likewise, MDDCs from the three groups of individuals exhibited similar levels of ovalbumin uptake and degradation (Fig 3B). Therefore MDDCs from HICs appear to have a similar intrinsic capacity to capture and process antigens when compared to cells from HDs and cARTs.

### C-type lectin receptors and syndecan-3 are more expressed on the surface of HICs’ MDDCs

**Fig 4:**
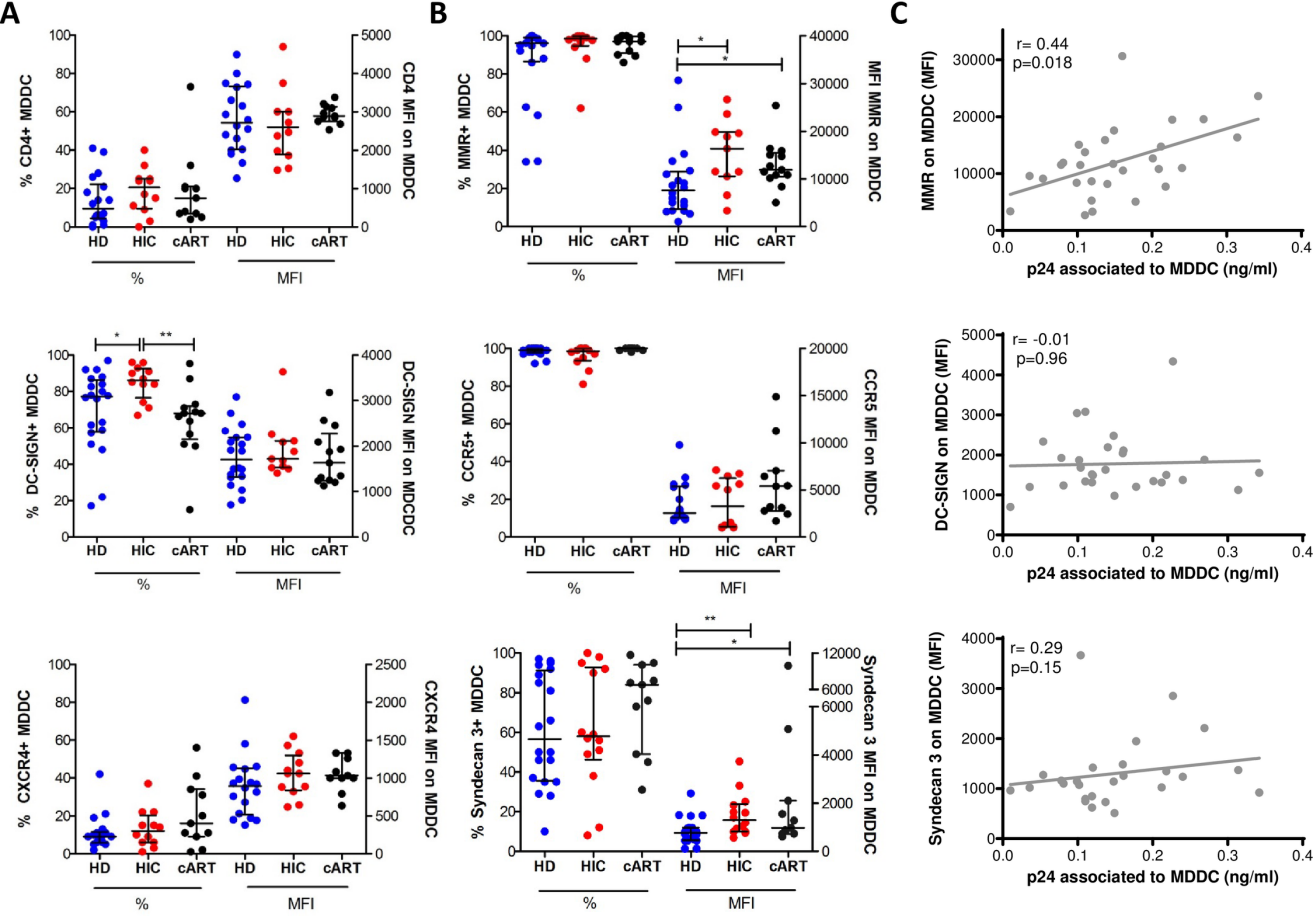
Expression of surface receptors on MDDC. **(A)** Expression levels of CD4, CXCR4 and CCR5 on MDDC from HDs (n = 11), HICs (n = 12) and cARTs (n = 18). **(B)** Idem for DC-SIGN, MMR and Syndecan-3 (HDs n = 19, HICs n = 14 and cARTs (n = 13). Each symbol represents one individual and horizontal lines represent the median ± interquartile for each group. * represents p<0.05; ** represents p<0.01. **(C)** Correlations between the levels of p24 captured by MDDC and their surface expression of MMR, DC-SIGN and Syndecan-3. Each symbol represents data obtained with cells from one patients (n = 29).

Our results suggest that the enhanced capacity of MDDCs from HICs to capture HIV-1 may be related to specific interaction of MDDCs with the HIV-1 particles. We therefore assessed the surface expression on MDDC from HICs, HDs and cARTs of different molecules that have been associated with HIV-1 capture. No difference was found in the expression of CD4, CCR5 or CXCR4 on MDDC from HICs, HDs and cARTs (Fig 4A). The expression of the Fcγ receptors (CD16, CD32 and CD64) or CD91 was not higher in the cells from HICs (S2 Fig). In contrast, we found that MDDCs from HICs expressed higher levels of MMR (p = 0.021) and Syndecan-3 (p = 0.006) compared to cells from HDs (Fig 4B), although not significantly higher than MDDCs from cARTs. In addition, the frequency of MDDCs with detectable levels of DC-SIGN was higher in MDDCs from HICs than in MDDCs from HDs (p = 0.035) or cARTs (p = 0.003). We found a correlation between the surface expression of MMR and the amount of p24 that the MDDCs captured in 29 subjects in whom both parameters were analyzed simultaneously (Fig 4C). We also found a tendency between the expression of syndecan-3 and the levels of p24. Overall, our results suggest that high expression of MMR, syndecan3 and DC-SIGN on the surface of MDDCs from HICs could synergize and cooperate with the HIV-1 receptors CD4 and CCR5 to efficiently capture HIV-1 particles.

## Discussion

In the present study we found that MDDC from HICs are less susceptible to HIV-1 infection than cells from healthy donors. In addition, our results show for the first time that MDDC from HICs capture greater amounts of HIV-1 than do MDDC from HD and cART patients.

The reduced susceptibility of MDDC from HICs to HIV-1 infection is in agreement with previous studies showing a reduced permissiveness to HIV-1 of CD4+ T cells and macrophages from the same patients [22–24]. Our results are consistent with a recent report of low infection level of primary DCs from HIC compared to cells from healthy donors [25]. Our findings suggest that an intrinsic resistance to HIV-1 infection is generalized to different cell subsets from HICs, strengthening the hypothesis that this feature contributes to the viral control in these individuals [22]. It has been recently proposed that an efficient sensing of HIV-1 by target cells could trigger IFN-I responses which would contribute to increase viral restriction in the cells and, in the case of DCs, enhance their capacity to activate HIV-specific T cell responses [26]. Martin-Gayo and colleagues recently found higher induction of type I IFN and interferon stimulated genes in primary DCs from HIC after HIV-1 infection [25]. We did not find detectable levels of IFN-α produced by cells from either HICs and HDs after infection, in accordance with the literature [21, 27]. Although it is still unclear whether the same mechanism(s) of restriction are responsible for relative resistance to HIV-1 infection of different cell types from HICs, we did not find evidences of increased IFN-α activity either in previous studies on CD4+ T cells and macrophages from HICs ([22] and not shown). The discrepancy between our results and the results by Martin-Gayo and colleagues could be due to the difference in the cell models analyzed (MDDC vs circulating BDCA1+ DC) as well as the way to measure IFN-I response (quantification of IFN-I in supernatants in our study vs intracellular mRNA levels).

Infection of DCs by HIV-1 causes different disturbances on these cells compromising their functions, including their capacity to stimulate CD8+ T cells [28, 29]. Based on our results we can therefore speculate that resistance of MDDC from HIC to HIV-1 infection may preserve their function and capacity to induce efficient CD8+ T cell responses. DCs are able to present HIV-1 antigens to CD8+ T cells either by processing intracellular protein stemming from HIV replication or by uptake of HIV-1 antigens from extracellular media. Interestingly, while MDDCs from cART patients seemed to capture HIV-1 particles less efficiently than cells from healthy donors, the MDDCs from HICs showed an enhanced capacity to bind HIV-1 when compared to cells from the two other groups of individuals. This efficient capture of HIV-1 by MDDCs from HICs is not explained by increased expression of CD4 or CCR5 on the cell surface. In contrast, we observed high expression of MMR, Syndecan-3 and, in particular, DC-SIGN on HIC MDDCs. These receptors are known to interact with carbohydrates on the surface of gp120 enhancing and stabilizing the capture of HIV particles by DCs [30–32]. The high expression of these receptors was not associated with a higher capacity of MDDCs from HICs to capture dextrans or ovalbumine. Although MMR-independent capture of these molecules may contribute to this lack of difference, these results suggest that MDDCs from HICs do not have a general higher capacity to capture and process antigens. High levels of MMR, DC-SIGN and Syndecan-3 may however act synergistically with other viral receptors on the surface of MDDCs such as CD4 and CCR5 to enhance HIV-1 capture, although we did not have enough cells to directly evaluate this possibility.

The route of antigen uptake, dictate the pathway for antigen processing and presentation. Interestingly, the involvement of both MMR and DC-SIGN in the capture of exogenous antigens promotes the cross-presentation process [33–37]. Thus, it is tempting to speculate that the higher expression of MMR, DC-SIGN and syndecan-3 on MDDCs from HICs might cooperate for HIV-1 uptake and could favor cross-presentation of HIV-1 antigens to CD8+ T cells. Increased binding of HIV-1 particle through MMR or DC-SIGN might enhance transmission of infection to CD4+ T cells [38, 39], however this effect might be limited in the case of HIV controllers due to the relative resistance of their CD4+ T cells to HIV-1 infection [22, 23]. The properties of DCs from HICs might also be relevant to the priming and activation of their HIV-specific CD4+ T cell responses, which have been shown to possess a highly efficient effector phenotype able to respond to limit amounts of antigens [40, 41]. Combined, the properties of DCs from HICs might protect them from HIV-1 infection, thereby preserving their functionality, while favoring the cross-priming of HIV-specific CD8+ T cell responses in a context of low antigen availability.

## Materials and Methods

### Subjects

HICs were enrolled from the ANRS CO21 cohort and were defined as patients infected by HIV-1 for ≥ 5 years who never received antiretroviral treatment and whose last 5 consecutive plasma HIV RNA values were < 400 copies/mL. HIV-infected patients on antiretroviral therapy with viral load < 50 RNA copies/mL for ≥ 6 months (cART) were recruited among patients followed at Hôpital Foch, Hôpital Européen Georges-Pompidou and Hôpital Saint-Louis in France. A summary of the characteristics of the HIV-1 patients included in the study can be found in the S2 Table. Blood samples from healthy HIV-seronegative donors (HD), were obtained from the Establissement Français du Sang (EFS) and Institut Pasteur plate-form Investigation Clinique et Accès aux Ressources Biologiques (ICAReB).

### Ethics Statement

All enrolled patients gave written informed consent to participate in the study. The CO21 HIV controller cohort is sponsored by the French National Agency for Research on AIDS and Viral Hepatitis (ANRS). The C021 CODEX cohort and this substudy protocol were approved by the ethics review committee of Ile de France VII and the institutional review board of Institut Pasteur.

### Generation of monocyte derived dendritic cells (MDDC)

Peripheral blood mononuclear cells (PBMC) were isolated from fresh whole blood by density gradient in lymphocyte separation medium (Eurobio, Abcys). Red blood cells were lysed by hypotonic shock. Monocytes (CD14+) were purified from PBMC by positive selection with antibody magnetic beads in a Robosep instrument (Stem cell Technology). Monocytes were differentiated into dendritic cells in presence of GM-CSF (1000 IU/ml) and IL-4 (1000 IU/ml) (R&D Systems) during 6–7 days.

### Infection of MDDC

Immature MDDC (10^5^) were pulsed in triplicate with HIV-1 BaL (10^−1,3^ MOI) during 4h at 37°C. The cells were then washed twice and cultured for 20 days. Every 3–4 days, culture supernatants were recovered and replenished with fresh medium. Viral replication was monitored in supernatants by p24 Elisa (Zeptometrix, Gentaur, France).

### HIV-1 capture

MDDC (10^5^) were pulsed in triplicate with HIV-1 BaL (2 ng of p24/10^5^ cells) in 100 μl of medium for 4 h at 37°C without spinoculation. Cells were then washed three times to eliminate free HIV particles and lysed. Cell-associated HIV-1 was quantified by Elisa p24 (Zeptometrix, Gentaur,France).

### Quantification of bioactive IFN-α

IFN-α activity in culture supernatants of infected DCs was determined, at different time points after infection, using an ultrasensitive functional assay based on protection of Madin-Darby bovine kidney (MDBK) cells against the cytolytic effect of vesicular stomatitis virus [42]. The titers were expressed in international units based on the reference standard for human IFN-alpha (G-023-901-527; NIH, Bethesda, MD).The test measures all subtypes of human IFN-α.

### Study of antigen uptake and degradation by MDDCs

To analyze the capacity of MDDCs to capture different antigens we measured the uptake of Alexa 488-labelled dextran and DQ ovalbumin. MDDCs (10^5^) were pulsed with 5 μg of Dextran-Alexa fluor 488 (Life Technologie) or DQ-Ovalbumine (Life Technologie) during 4 h at 37°C or 4°C. Cells were washed twice, stained during 15 min at 4°C with anti-HLA-DR-ECD (Beckman Coulter) and anti-CD11c-V450 (BD Biosciences) antibodies and then fixed with 2% of paraformaldehyde. Cells were analyzed with a LSR II flow cytometer (Becton Dickinson). Cells incubated at 4°C were used as negative control.

### Expression of receptors on the surface of MDDCs

MDDC were incubated 15 min at RT with CD11c-V450 (clone mP9, BD), HLA-DR-ECD (clone Immu-357, BC) and with different panel of antibodies to assess the expression level of different receptors. Panel I: CD4-FITC (clone L120, BD), CCR5-APC (clone 3A9, BD), CXCR4-PE (clone 12G5, BD); Panel II: MMR-FITC (clone19.2, BD), DC-SIGN-PerCp (clone, R&D), CD91 (clone A2MR-α2, BD), Syndecan 3 PerCp Cy5.5 (clone MI15, BD); and Panel III: CD16-APC (Clone B73.1, BD), CD32-PE(Clone 3D3, BD), CD64-FITC (Clone 10.1, BD). After staining, cells were washed twice and resuspended in PBS containing 2% of paraformaldehyde and 1% of fetal calf serum. MDDC were confirmed as HLA-DR and CD11C positive cells. Data were acquired on a LSR II cytometer and analysed using DIVA software (both from BD Biosystems).

### Statistical analyses

Data were described by medians and interquartile ranges (IQR) for continuous variables Nonparametric ANOVA or Mann-Whitney tests were used to compare datasets between groups. The Spearman’s non parametric correlation was used to estimate the association of two continuous variables of interest.

All statistical analyses were done with GraphPad Prism 5.03 software (GraphPad software, La Jolla, USA). In 2-tailed tests, P values of 0.05 or lower were considered significant.

## Supporting Information

S1 Appendix the ANRS CODEX-CO21 cohort study group, list of clinical contributors. (DOC)

S1 Fig Lack of correlation between HIV-1 BaL capture and peak of replication in MDDC from HDs n = 17 (left) and HICs n = 20 (right).
(PDF)

S2 Fig Expression level of FCγreceptors and CD91 on MDDC from HDs (n = 11), HICs (n = 12) and cARTs (n = 18). Each symbol represents one individual and horizontal lines represent the median ± interquartile for each group.
(PDF)

S1 Table IFN-I quantification by functional test in supernatants of HIV-1 infected MDDC. (DOCX)

S2 Table Characteristics of HIV-1 infected patients included in the study. (DOCX)
