# Supplementary material for: Dendritic Cells from HIV Controllers Have Low Susceptibility to HIV-1 Infection In Vitro but High Capacity to Capture HIV-1 Particles
Source: PLoS One. 2016 Aug 9;11(8):e0160251. doi: 10.1371/journal.pone.0160251 (PMC4978443; doi:10.1371/journal.pone.0160251)
Supplement: S1 Appendix — (DOC) [file pone.0160251.s001.doc]

**S1 Appendix**

**The ANRS CODEX-CO21 cohort study group:**

Dr Jean-Pierre Faller, Mme Patricia Eglinger, Service des Maladies Infectieuses, CH de Belfort-Montbéliard, Belfort. Pr Pascal Roblot, M David Plainchamp, Service de Médecine Interne, CHU Poitiers-La Milétrie, Poitiers. Dr Hugues Aumaître, Mme Martine Malet, Service des Maladies Infectieuse et Tropicales, CH de Perpignan, Perpignan. Dr Christine Rouger, Pr Gérard Rémy, Melle Kmiec Isabelle, Service des Maladies Infectieuses, CHU Reims-Hôpital Robert Debré, Reims. Dr Jean-Luc Delassus, Service de Médecine Interne, CHI Ballanger, Aulnay Sous-Bois. Dr Alain Devidas, Service d’Hématologie, CH Sud-Francilien -Hôpital Gilles de Corbeil, Corbeil - Evry. Dr Eric Froguel, Mme Sylvie Tassi, Service de Médecine Interne-Maladies Infectieuses, CH de Marne la Vallée, Jossigny. Dr Philippe Genet, Mme Juliette Gerbe, Service Hématologie-Immunologie, Centre Hospitalier Victor Dupouy, Argenteuil. Pr Olivier Patey, Mr Richier Laurent, Service des Maladies Infectieuses et Tropicales, CHI Villeneuve Saint Georges, Villeneuve Saint Georges. Dr Marie-Christine Drobacheff, Dr Aurélie Proust, Service de Dermatologie, Hôpital Saint-Jacques, Besançon. Dr Helder Gil, Service de Maladies Infectieuses et Tropicales, Besançon. Dr Laurence Gérard, Pr Eric Oksenhendler, Service d'Immuno-pathologie, Hôpital Saint Louis, Paris. Pr Frédéric Lucht, Mme Véronique Ronat, Service de Maladie Infectieuse, Hôpital Bellevue, Saint Etienne. Pr Michel Dupon, Dr Hervé Dutronc, Mme Séverine Le Puil, Service des Maladies Infectieuses, CHU- Hôpital Pellegrin, Bordeaux. Pr Jean-Luc Schmit, Mme Nathalie Decaux, Service de pathologies infectieuses, CHU- Hôpital Nord, Amiens. Pr Jean-Michel Molina, Dr Caroline Lascoux, Mme Sylvie Parlier, Service de Maladies Infectieuses et Tropicales, Hôpital Saint Louis, Paris. Dr Jean-Pierre BRU, Mme Gaëlle Clavere, Service des Maladies Infectieuses, Centre Hospitalier Annecy, Annecy. Pr Olivier Lambotte, Pr Jean-François Delfraissy, Pr Cécile Goujard, Mme Katia Bourdic, Service de Médecine Interne, Hôpital de Bicêtre, Le Kremlin Bicêtre. Pr Jean-François Bergmann, Mme Maguy Parrinello, Service de Médecine Interne A, Hôpital Lariboisière, Paris. Dr Gilles Pichancourt, Service Hématologie, Hôpital Henri Duffaut, Avignon. Dr Yves Welker, Service de maladies Infectieuses, CHI de Poissy-Saint Germain en Laye, Saint Germain en Lay. Dr Alain Lafeuillade, Mme Philip Gisèle, Service d’Infectiologie, CHITS Hopital Sainte Musse, Toulon. Pr Christophe Rapp, Melle Lerondel, Service des Maladies Infectieuses, Hôpital d'Instruction des Armées Bégin, Saint Mandé. Dr Pierre de Truchis, Mme Huguette Berthe, Département de Médecine Aigue Spécialisée, Hôpital Raymond Poincarré, Garches. Dr Vincent Jeantils, Mme Fatouma Mchangama, Unité de Maladies Infectieuses, Hôpital Jean Verdier, Bondy. Pr. Daniel Vittecoq, Mme Claudine Bolliot, Service des Maladies Infectieuses, Hôpital de Bicêtre, Le Kremlin Bicêtre. Dr Paul Henri Consigny, Mme Fatima Touam, Consultation de Maladies Infectieuses, Centre Médical de l’Institut Pasteur, Paris. Pr Gilles Pialoux, Mme Sophie le Nagat, Service des Maladies Infectieuses, Hôpital Tenon, Paris. Pr Olivier Bouchaud, Mme Patricia Honoré, Service de Médecine Interne et Endocrinologie, Hôpital Avicenne, Bobigny. Pr François Boué, Mme Mariem Raho-Moussa, Service de Médecine Interne, Hôpital Antoine Béclère, Clamart. Pr Laurence Weiss, Dr Lio Collias, Service d'Immunologie Clinique, HEGP, Paris. Pr Dominique Salmon-Céron, Mme Marie-Pierre Pietri, Service de Médecine Interne et centre références Maladies Rares, Hôpital Cochin, Paris. Dr Zucman, Pr Olivier Blétry, Mme Dominique Bornarel, Service de Médecine Interne, Hôpital Foch, Suresnes. Dr Emmanuel Mortier, Mme Zeng Feng, Service de Médecine Interne, Hôpital Louis Mourier, Colombes. Pr Jean-Daniel Lelièvre, Service d'Immunologie Clinique, Hôpital Henri Mondor, Créteil. Pr Christine Katlama, Mme Yasmine Dudoit, Service des Maladies Infectieuses, Hôpital Pitié-Salpêtrière, Paris. Dr Anne Simon, Mme Catherine Lupin, Service des Maladies Infectieuses, Hôpital Pitié-Salpêtrière, Paris. Pr Pierre-Marie Girard, Mme Michèle Pauchard, Service des Maladies Infectieuses, Hôpital saint Antoine, Paris. Dr Sylvie Abel, Dr André Cabié, Service de Maladies Infectieuses et Tropicales, Hôpital Pierre Zobda-Quitman, Fort de France, Martinique. Dr Pascale Fialaire, Dr Jean-Marie Chennebault, M Sami Rehaiem, Service des Maladies Infectieuses et Tropicales, CHU Angers, Angers. Dr Luc de Saint Martin, Dr Perfezou, M Jean-Charles Duthe, Service de Pneumologie, CHU de Brest, Brest. Pr Philippe Morlat, Mme Sabrina Caldato, Service de Médecine Interne, CHU- Hôpital Saint André, Bordeaux. Pr Didier Neau, Mme Séverine LE Puil, Service des Maladies Infectieuses A, CHU- Hôpital Pellegrin, Bordeaux. Pr Pierre Weinbreck, Dr Claire Genet, Service des Maladies Infectieuses, CHU de Limoges, Limoges. Dr Dr Djamila Makhloufi, Mme Florence GARNIER, Service d'Immunologie clinique, HCL- Hôpital Edouard Herriot, Lyon. Dr Isabelle Poizot-Martin, Dr Olivia Fauche, Mme Alena Ivanova, Service Hématologie- Cisih, Hôpital Sainte Marguerite, Marseille. Pr Patrick Yeni, Dr Sophie Matheron, Mme Godard Cyndi, Service des Maladies Infectieuses, Hôpital Bichat Claude Bernard, Paris. Pr François Raffi, Mr Hervé Hüe, Service de Médecine Interne, Hôpital de l’Hôtel Dieu, Nantes. Dr Philippe Perré, Service de Médecine Interne post-Urgence, Centre Hospitalier Départemental, La Roche sur Yon. Pr Pierre Marie Roger, Mme Aline Joulie, Service des Maladies Infectieuses, CHU- Hôpital l’Archet, Nice. Pr Éric Rosenthal, Service Médecine Interne, CHU- Hôpital l’Archet, Nice. Pr Christian Michelet, Dr Faouzi Souala, Mme Maja Ratajczak, Service des Maladies Infectieuses, CHU-Hôpital Pontchaillou, Rennes. Dr Marialuisa Partisani, Mme Patricia Fischer, HUS-Hôpital Civil, Strasbourg. Pr Louis Bernard, Mme Pascale Nau, Service des Maladies Infectieuses, CHRU- Hôpital Bretonneau, Tours. Pr Bruno Marchou, Mme Florence Balsarin, Service des Maladies Infectieuses, CHU-Hôpital Purpan, Toulouse. Pr Renaud Verdon, Mr Philippe Feret, Service des Maladies Infectieuses, CHU- Hôpital de la Côte de Nacre, Caen. Dr Christine Jacomet, Service des maladies Infectieuse, CHU Gabriel Montpied, Clermont Ferrand. Dr Lionel Piroth, Mme Sandrine Gohier, Service de Maladies Infectieuses et Tropicales, CHU-Hôpital du Bocage, Dijon. Dr Pascale Leclercq, Mme Gerberon, Service Médecin Aigue spécialisée, CHU-Hôpital Albert Michallon, Grenoble. Dr Agnés Meybeck, Dr Raphaël Biekre, Service des Maladies Infectieuses, CH- Hôpital Gustave Dron, Tourcoing. Pr Thierry May, Mme Bouillon, Service de maladies Infectieuses et tropicales, CHU Nancy, Nancy. Pr François Caron, Dr Yasmine Debab, M David Theron, Service de maladies Infectieuses et tropicales, CHU- Hôpital Charles Nicolle, Rouen. Dr Patrick Miailhes, M Stanislas Ogoudjobi, Service de Maladies Infectieuses et Tropicales, HCL- Hôpital de la Croix Rousse, Lyon. Pr Patrick Mercié, Service Tropicales, CHU- Hôpital Saint André, Bordeaux. Dr Marc Gatfosse, Service de Médecine Interne, CH René Arbeltier, Coulommiers.

Dr Martin Martinot, Mm Anne Pachart, service de maladies infectieuses-Médecin Interne, Hôpitaux Civils de Colmar, Colmar. Dr Patrice Poubeau, Service de Pneumo-phtisiologie, Centre Hospitalier Sud Réunion - Hôpital de St Pierre, Saint Pierre, La Réunion. Dr Agnès Uludag, Service de Médecine Interne, Hôpital Beaujon, Clichy. Dr Philippe Arsac, Mme Lydia Bouaraba, Service de Médecine Interne, CHR Orléans- Hôpital Porte Madeleine, Orléans. Dr Isabelle De Lacroix Szmania, M Laurent Richier, Service des Médecine Interne, Centre Hospitalier Intercommunal, Créteil. Dr Vincent Daneluzzi, Service de Médecine A, CASH - Hôpital Max Fourestier, Nanterre. Dr Elisabeth Rouveix, Service de Médecine Interne 2, Hôpital Ambroise Paré, Boulogne. Dr Geneviève Beck-Wirth, Service d'Hématologie Clinique VIH, Centre Hospitalier de Mulhouse, Mulhouse. Dr Philippe Romand, Service de Pneumologie, CHI Les Hôpitaux du Léman, Thonon les Bains. Dr Laurent Blum, Mme Martine Deschaud, Service Médecine-Gastroentérologie, Centre hospitalier René Dubos, Pontoise. Dr Christophe Michau, Service de Médecine Interne, Centre Hospitalier de Saint Nazaire, Saint Nazaire. Dr Christian Bernard, Mme Florence Salaun, Service de Médecine Interne, CHR Metz Thionville Hôpital Notre Dame de Bon Secours, Metz. Dr Philippe Muller, Service de Dermatologie, Hôpital Beauregard, Thionville. Dr Yves Poinsignon, Service de Médecine Interne, Hôpital Prosper Chubert, CHBA, Vannes. Dr Annie Lepretre, Mme Martine Deschaud, Service de Médecine Interne, Hôpital Simone Veil, Eaubonne. Dr Thierry Lambert, Consultation d’Hématologie, CHU de Bicêtre, Le Kremlin Bicêtre. Dr Laurent Hocqueloux, Mme Barbara de Dieulevault, Service de Maladies Infectieuses et Tropicales, Hôpital Orléans la Source, Orléans. Dr Patrick Philibert, Mme Mame Penda Sow, Consultation de Médecine Interne, Hôpital Européen Marseille, Marseille. Pr Albert Sotto, Mme Doncesco, Service des Maladies Infectieuses et Tropicales, CHU Caremeau, Nîmes. Dr Jean-Paul Viard, Mme Agnés Cros, Centre de diagnostic et de thérapeutique, Hôpital Hotel Dieu, Paris. Dr Marc De Lavaissiere, Service Médecine Interne, CHG de Montauban, Montauban. Dr Pascale Perfezou, M Jean Charles DUTHE, Service de Pneumologie, CH de Cornouaille-Hôpital Laennec, Quimper. Dr Catherine Gaud, Service Immunologie Clinique, Centre Hospitalier Félix Guyon, Ile de la Réunion. Dr Mathilde Aurore Niault, Mme Virginie Mouton- Rioux, Service d'hématologie, maladie Infectieuses, CH Bretagne Sud, Lorient. Dr Jean –Philippe Talarmin, M Jean Charles Duthé, Service Médecine Interne, CH de Cornouaille-Hôpital Laennec, Quimper. Dr Dupont Mathilde, M Stéphane Natur, Service des Maladies Infectieuses et Tropicales, CH Saint Malo, Saint Malo. Dr Hikombo Hitoto, M Ali Mahamadou Ibrahim, Service de Maladies Infectieuses et tropicales, Centre Hospitalier Le Mans, Le Mans.
