# Supplementary figures and images for: Dendritic Cells from HIV Controllers Have Low Susceptibility to HIV-1 Infection In Vitro but High Capacity to Capture HIV-1 Particles
Source: PLoS One. 2016 Aug 9;11(8):e0160251. doi: 10.1371/journal.pone.0160251 (PMC4978443; doi:10.1371/journal.pone.0160251)

## S1 Figure

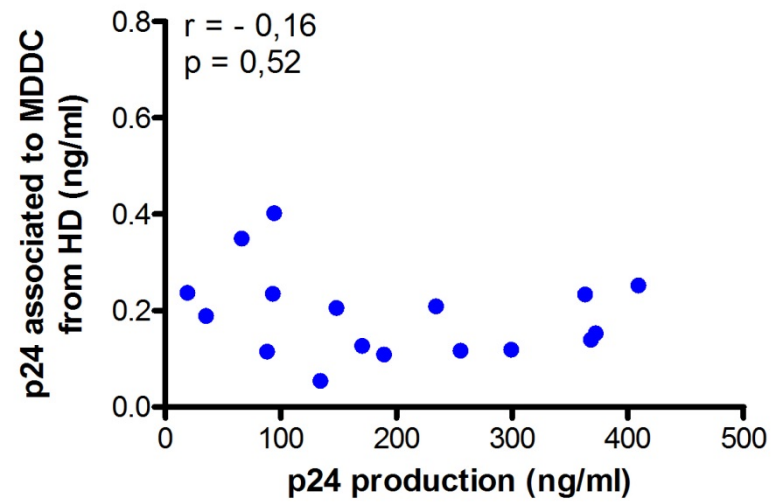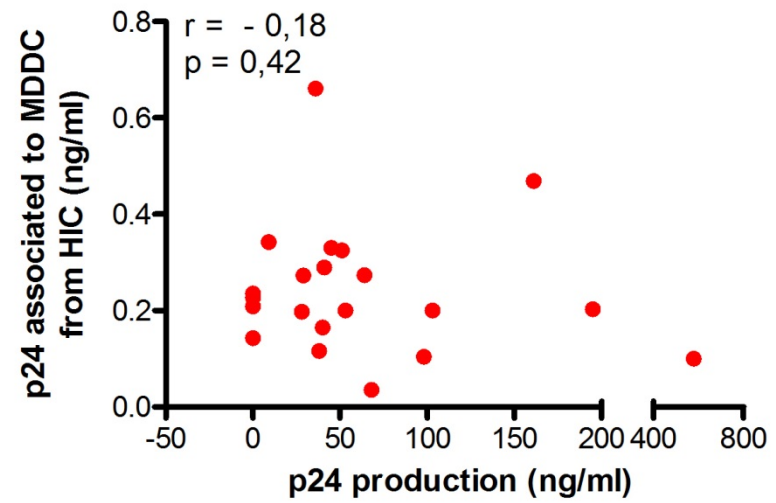

Supplement: S1 Fig — Lack of correlation between HIV-1 BaL capture and peak of replication in MDDC from HDs n = 17 (left) and HICs n = 20 (right). (PDF) [file pone.0160251.s002.pdf]

S2 Figure

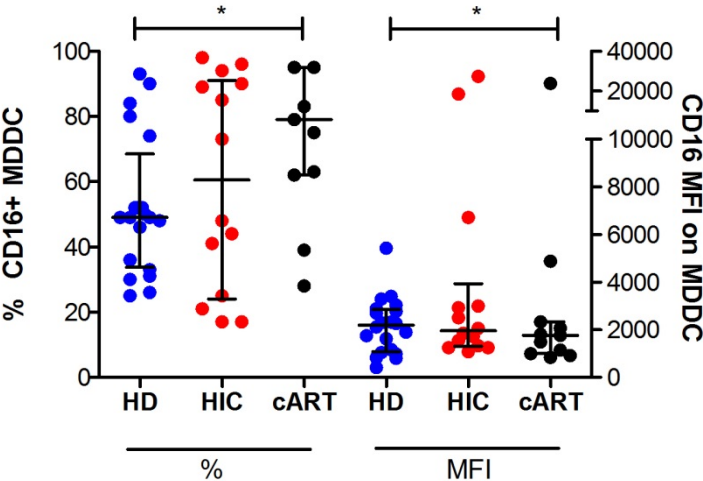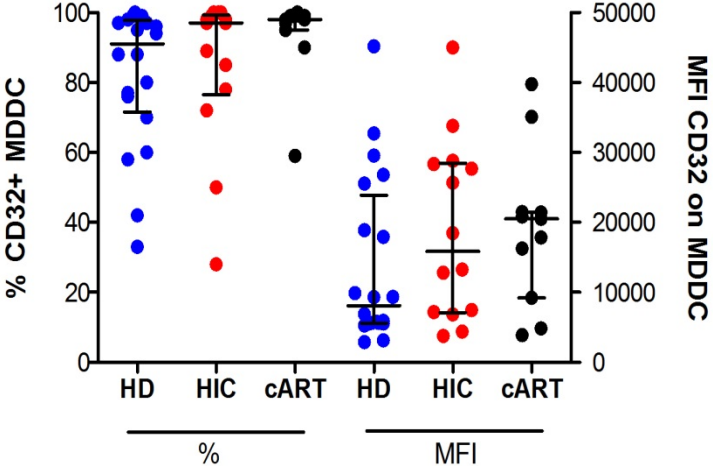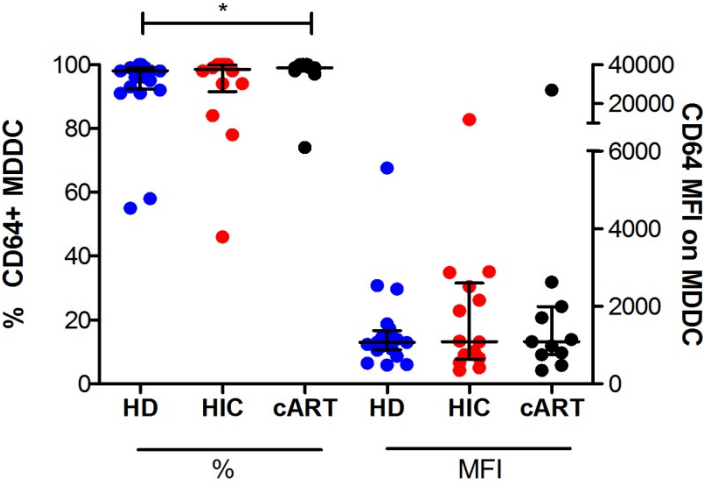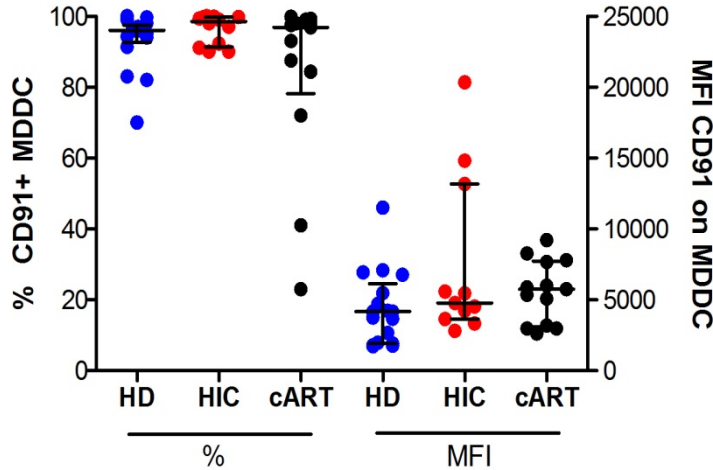

Supplement: S2 Fig — Each symbol represents one individual and horizontal lines represent the median ± interquartile for each group. (PDF) [file pone.0160251.s003.pdf]
