# Supplementary material for: Dendritic Cells from HIV Controllers Have Low Susceptibility to HIV-1 Infection In Vitro but High Capacity to Capture HIV-1 Particles
Source: PLoS One. 2016 Aug 9;11(8):e0160251. doi: 10.1371/journal.pone.0160251 (PMC4978443; doi:10.1371/journal.pone.0160251)
Supplement: S1 Table — (DOCX) [file pone.0160251.s004.docx]

**S1 Table.** IFN‐I quantification by functional test in day 13 supernatants of HIV‐1 infected MDDC

| Day 13 | | **p24( ng/ml)** | **IFN-I (IU/ml)** |
| --- | --- | --- | --- |
| **HIC** | 17003 | 28 | UD |
|  | 114002 | 36 | UD |
|  | 34002 | 53 | UD |
|  | 34014 | 5 | UD |
|  | 13001 | 57 | UD |
|  | 62012 | 62 | UD |
|  | 196004 | 6 | UD |
|  | 93003 | 9 | UD |
|  | 171003 | 28 | UD |
|  | 56011 | 51 | 24 |
|  | 62014 | 45 | UD |
|  | 34015 | 53 | UD |
|  | 34017 | 550 | UD |
|  | 28001 | 0 | UD |
|  | 81001 | 162 | UD |
|  | 86003 | 296 | UD |
|  | 63011 | 98 | UD |
|  | 92005 | 9 | UD |
|  | 198001 | 41 | UD |
| **HD** | EFS167 | 187 | UD |
|  | EFS168 | 144 | UD |
|  | EFS 170 | 222 | UD |
|  | EFS 171 | 137 | 4.3 |
|  | EFS 346 | 134 | UD |
|  | EFS 300 | 344 | 5.4 |
|  | EFS 297 | 376 | UD |
|  | EFS377 | 160 | 16.5 |
|  | EFS 364 | 19 | UD |
|  | EFS 365 | 88 | UD |
|  | EFS 361 | 35 | UD |
|  | EFS 352 | 223 | UD |
|  | EFS 353 | 198 | UD |
|  | EFS 338 | 409 | UD |
|  | EFS 346 | 134 | UD |
