# Supplementary material for: Dendritic Cells from HIV Controllers Have Low Susceptibility to HIV-1 Infection In Vitro but High Capacity to Capture HIV-1 Particles
Source: PLoS One. 2016 Aug 9;11(8):e0160251. doi: 10.1371/journal.pone.0160251 (PMC4978443; doi:10.1371/journal.pone.0160251)
Supplement: S2 Table — (DOCX) [file pone.0160251.s005.docx]

**Supplementary Table 2.** Characteristics of HIV-1 infected patients included in the study

|  | HIV controllers | cART patients |
| --- | --- | --- |
| n | 52 | 25 |
| Gender | F:25 M: 27 | F: 5 ; M : 19 |
| Age | 47 [42-54] | 52 [48-59] |
| CD4+ T cells/mm3 | 793 [638-966] | 600 [485-758] |
| Viral load (RNA copies/ml) | <40 [<20-53] | <40 [<20-<40] |
